# Adherence to treatment in patients with borderline personality disorder (BPD) with gynecological cancer

**DOI:** 10.1192/j.eurpsy.2025.1904

**Published:** 2025-08-26

**Authors:** M. Garza, A. Alcorta, C. Martinez, A. Cantú

**Affiliations:** 1 Hospital Universitario Dr José Eleuterio González, Monterrey, Mexico; 2 Psicooncología, Medicina de Enlace y Cuidados Paliativos, Hospital Universitario Dr José Eleuterio González, Monterrey, Mexico

## Abstract

**Introduction:**

The presence of borderline personality disorder (BPD) in cancer patients can be a risk factor for adherence to oncological treatment.

**Objectives:**

To analyze problems related to adherence to treatment of patients with borderline personality disorder (BPD) in comorbidity with gynecological cancer.

**Methods:**

Clinical histories and subsequent interviews of patients (“A” and “B”), both 25 years of age. “A” with a diagnosis of Cervical Cancer in 2022, without adherence to treatment, irritable, unmotivated, with emotional instability in her relationships, chronic emptiness, impulsivity, risky sexual behaviors, self-harm, and suicidal thoughts. B, diagnosed with endometrial cancer in January 2023, presents treatment adherence with extreme fear of abandonment, irritability, anhedonia, anxiety, low self-esteem, impulsivity, and suicidal ideas. Both receive psychotherapy at the Psychooncology and Palliative Care clinic. Scales from the General Health Questionnaire (GHQ-28) and McLean Screening Instrument for Borderline Personality Disorder were applied.

**Results:**

Anxiety, lack of control, and rebellion manifested A’s lack of adherence. B’s “adherence” is redefined as separation anxiety, currently deposited in her partner and the oncology team. They evoked physical and sexual violence experienced since childhood and drug abuse, which influenced her anger and attitude towards cancer treatment for being “captives” of the disease. Thus, she denied A her dependency needs and B her fear of her partner’s abandonment and her suicidal thoughts.

**Conclusions:**

Internal conflicts are expressed through self-destructive behavior: addressing her fears allowed her to continue treatment. Psychotherapy limited his judgment, cognitive, and reasoning lapses. They mentalized stories of abuse, early violence, and behaviors that limited their quality of life before and after cancer, improving their treatment adherence.

**Disclosure of Interest:**

None Declared

